# Frailty status and associated factors among older PLHIV in Southern Ethiopia

**DOI:** 10.1371/journal.pone.0284376

**Published:** 2023-04-24

**Authors:** Endrias Markos Woldesemayat, Natalie St Clair-Sullivan, Andargachew Kassa, Taye Gari, Keneni Gutema, Nana Chea, Kindie Woubshet, Netsanet Bogale, Amare Assefa, Jaime Vera

**Affiliations:** 1 College of Medicine and Health Sciences, Hawassa University, Hawassa, Ethiopia; 2 Department of Global Health and Infection, Brighton and Sussex Medical School, University of Sussex, Brighton, United Kingdom; 3 College of Health Sciences, Jima University, Jima, Ethiopia; Ege University Faculty of Medicine: Ege Universitesi Tip Fakultesi, TURKEY

## Abstract

**Background:**

Studies addressing frailty are limited in the global south, including Ethiopia. We estimated the prevalence of frailty and associated factors among older people living with HIV (PLHIV) attending a large Comprehensive Specialized Hospital in southern Ethiopia.

**Methods:**

A systematic sample of 187 PLHIV and 187 HIV-negative controls > 50 years old were recruited between October 1 and November 30, 2021. Data on socio-demographic, behavioural and clinical characteristics were collected using a structured questionnaire. Frailty assessments were completed using the brief frailty instrument (B-FIT-2), which consists of 6 components. Scoring 5–6 points was frail, 2–4 points were pre-frail and below 2 was considered as non-frail. Logistic regression model was used to measure association between variables.

**Results:**

Median (IQR) age was 53 (50, 80) for PLWH and 59 (55–66) for controls. Prevalence of frailty was 9.1% for PLHIV Versus 5.9% for controls. A significant proportion of PLHIV was pre-frail; 141 (75.4%) compared to controls 110 (58.8%). Pre-frailty status was associated with HIV diagnosis (adjusted odds ratio (aOR) 4.2; 95% CI 1.8–9.9), low age (aOR 0.3; 95% CI 0.1–0.6), lower educational attainment (aOR 2.2; 95% CI 1.0–4.9), being farmer (aOR 3.2; 95% CI 1.0–10.2) and having high or low body mass index (BMI) (aOR 11.3; 95% CI 4.0–25.8). HIV diagnosis (aOR 9.7; 95% CI 1.6–56.8), age (aOR 0.2; 95% CI 0.1–0.7), lower educational attainment (aOR 5.2; 95% CI 1.5–18.2), single status (aOR 4.2; 95% CI 1.3–13.6), farmer (aOR 19.5; 95% CI 3.5–109.1) and high or low BMI (aOR 47.3; 95% CI 13.8–161.9) predicted frailty.

**Conclusion:**

A high proportion of frailty and pre-frailty was observed in a cohort of older PLHIV attending care in Southern Ethiopia. Future research should focus on interventions targeting factors associated with frailty.

## Introduction

As the life expectancy of people living with HIV (PLHIV) is improving, they experience a disproportionate amount of comorbidities and premature onset of geriatric syndromes, such as frailty [1, 2]. Frailty is a marker of vulnerability which is becoming more relevant among PLHIV as the population ages [3]. Frailty causes diminished physiologic reserve, increased vulnerability to stressors and higher risk of falls and comorbidity index [4]. It predisposes individuals to major adverse clinical outcomes, including hospitalization, institutionalization, disability and death of older adults, as well as lowering quality of life for PLHIV [4, 5].

The Fried Frailty Phenotype (FFP) has previously been used to estimate frailty in community-dwelling non-HIV older adults in rural Tanzania [6] and examines 5 domains; weakness, slow walking speed, unintentional weight loss, low physical activity and exhaustion. Additionally, the Brief Frailty Instrument for Tanzania (B-FIT) was developed from a longitudinal cohort study in North-eastern Tanzania of community-dwelling people aged 70 and over, and modelling was used to identify Tanzania-specific frailty markers [7]. The Clinical Frailty Scale, a pictograph with a short clinical description widely-used in high-income countries for identification of frailty in older adults [8]. There is no published research regarding its use in SSA.

Prevalence of frailty has been reported in several cohorts of PLHIV. In Colorado, USA 6% were found to be frail while 38% were pre-frail [9]. In Spain, the prevalence of pre-frailty and frailty were 39.1% and 4.4%, respectively [4]. In another study, the proportion of mild, moderate, and severe frail elderly patients in a cohort study in were 20%, 50% and 30%, respectively [10]. There have been a few previous studies of frailty among PLHIV reported in Sub-Saharan Africa (SSA). However both studies used the FFP as a measure of frailty. The study in South Africa reported a prevalence rate of 19.4% [2] and in Senegal the prevalence was 3.5% [11]. A recent paper by Bristow et al. [12], using the B-FIT 2 metric, showed a remarkably low prevalence of frailty of 0.68% among PLHIV over 50 years old in northern Tanzania. Neurocognitive impairment, depressive and/or insomnia symptoms, and gender were factors significantly correlated with frailty [1, 4, 13]. Other studies report that frailty increased with lower education, older age, initial efavirenz, smoking, obesity and lower current CD4 count [2, 9].; while physical activity reduced the risk of frailty [9].

A majority of frailty studies are conducted in the global north and for the few studies conducted in the global south findings differ depending on the geographical setting. For example factors such as older age and lower CD4 level in South Africa, a relatively rich country in Africa [2] are different from the factors in Ethiopia (advanced HIV and malnutrition) [14]. We have no data from South Ethiopia where some specific factors like nutritional status might be associated with frailty.

Identifying the level of frailty and associated factors among aging PLHIV will help us to design appropriate interventions to prevent frailty [15]. Data on frailty among PLHIV in sub-Saharan Africa (SSA) countries is scarce and to the best of our knowledge, there is no report on frailty among PLHIV in Southern Ethiopia. Therefore, in this study we aim to determine the prevalence and factors associated with frailty status and identify the association between clinical variables and individual components of frailty measures among older PLHIV at Hawassa University Comprehensive Specialized Hospital (HUCSH).

## Methods

### Study design and setting

We carried out institution based cross-sectional study among PLHIV and HIV-negative controls receiving health care at HUCSH. Hawassa town is the capital of both the Sidama Region and the Southern Nations and Nationalities Peoples Region (SNNPR) in Ethiopia. The hospital provides comprehensive health care for patients living with HIV and chronic non-communicable chronic diseases, which includes diabetes mellitus, asthma, hypertension and heart diseases. The anti-retroviral therapy (ART) unit in the hospital was established 15 years ago and currently provides care for over 2500 PLHIV.

### Study population and sample

PLHIV attending the ART unit at HUCSH between October 1 and November 30, 2021 was considered as the sources population for the exposed group (cases). During the same period, patients that came for chronic diseases follow-up care, who were HIV negative were considered as a source population for the non-exposed group (controls). Patients 50 years and over were included in the study. We recruited every seventh case among HIV negative eligible patients attending chronic diseases on-going care and every third PLHIV among the cases. Patients with mental illness and patients below the age of 50 years were excluded from the study.

The sample size was calculated using Openepi statistical software [16]. We used the following assumptions to calculate the sample size. A prevalence of frailty (p) among PLHIV of 54.9% which was reported in Indonesia [13], a 40% prevalence of frailty among non-HIV patients, 95% level of significance, 80% power and a 1:1 ratio of Unexposed/Exposed were considered. Based on these assumptions, the total sample size calculated was 187 for PLHIV and 187 for the comparison group (HIV non-infected controls). During data collection, we randomly selected the first participant from PLHIV who came to receive care and then we recruited every 3^rd^ person to be included in the exposed group. For the non-exposed group, at the first day of data collection, among participants that came to receive NCD care, we randomly identified the first participant and then every 7^th^ patient were selected to be included for the non-exposed group until we achieved the required sample size.

### Variables

Exposure measures considered in the study were socio-demographic characteristics such as gender, age, weight, height, marital status, education, occupation, residence and household income. Other clinical factors such as time of HIV diagnosis, duration on ART, ART adherence, comorbidities, CD4 count, and Haemoglobin level were also part of our exposure assessment. Viral load was not done regularly at HUCSH and therefore is not a variable in the report (read more on S1 File). The outcome measure considered in the study was frailty (being frail or pre-frail).

### Frailty assessment

Although the Fried Frailty Phenotype (FFP) is a commonly used non-specialist frailty assessment method in which you get objective data on weakness and walking speed [1, 4, 10, 13, 15], it is logistically difficult and time consuming. Also, it does not assess cognitive impairment, which is a key factor in frailty assessment. Therefore, we chose to use the brief frailty screening tool (BFIT-2) screening tool. The BFIT-2 is an updated version of a recently developed frailty metric, intended to be used by non-specialists to screen for frailty in older persons living in Sub-Saharan Africa (SSA). This tool addresses both the cognitive (the IDEA cognitive screen) [17] and functional (adapted Barthel Index) [18] screen. The BFIT screening tool is validated by a study in Tanzania [19] and has questions addressing cognition and physical function (including mobility, activity of daily living (ADLs) and continence), depression, social support, nutrition and sensory impairments which are relevant for the Ethiopian population. Nutritional assessment considered in this study was measurement of the BMI. Joining in social activities and distance vision impairment were also additional variables included in the tool. The B-FIT screen score has 0 to 6 levels. A score with cut-off values 0–1 was considered as non-frail, while 2–4 (pre-frail) and 5–6 (frail) [20]. This was evaluated based on the scores obtained for each criteria found in the B-FIT screening tool. For the B-FIT tool components there are a set of questions, and in each component a score above the average was considered as having a problem in the measured component. A point was given for each component score, then the sum of these values was considered to determine the presence of frailty.

Cognitive function was assessed by using the IDEA cognitive screen [17, 21]. Physical function was assessed by measuring continence, weakness, exhaustion, mobility, disability, physical activity and falls [22]. For this we used the Barthel index; falls, hand grip strength (HGS), timed-up-and-go (TUG) test, Gait speed. Absence of the required value in at least one of these characteristics was considered as having a problem in physical function. Mobility was assessed by measuring the TUG test and gait speed over 4.5 meters [23]. Falls were assessed through self-reported number of falls, both in the last year and in the last two months and weakness was measured using HGS. Although peak expiratory flow (PEF) is an important parameter in assessment of weaknesses in frailty, it was not measured because of COVID risks. Exhaustion was measured by two self-reported questions; ‘I felt that everything I did was an effort’ and ‘I could not get going’ [24]. Nutritional status was assessed using Body Mass Index (BMI). BMI value lying between 18.5 and24.9 kg/ht^2^ was considered as normal. Depression was assessed using the BECKS depression inventory which involves 22 questions [25]. A score of 1–14 was considered as normal (minimal depression). However, scores above 14 were considered as having varying degrees of depression [26]. Morbidity was assessed based on self-reported presence of diseases like anaemia, arthritis, benign prostatic hyperplasia, cancer and etc. Sensory impairments such as hearing loss and sight problems were assessed and included in the morbidity. We also assessed self-reported social support.

### Data collection techniques

A structured questionnaire was used to collect data (see supplementary material). The questionnaire was developed based on the listed variables in the above section. Data on clinical variables were collected through interviews and record review from October 1 and November 30, 2021. Primary data on socio-demographic characteristics and frailty related variables were obtained through interview. Besides, the listed variables in the above section, assessment of patients history of falls, comorbidities, cognition (IDEA cognitive screen), physical function related questions, nutrition, mood, perceived health, sensory impairment (vision and hearing), poly-pharmacy and social support was conducted. The questionnaire was first prepared in English and then translated to Amharic and then translated back to English for checking consistency in meanings. Enumerators (nurses with B. Sc. degree from ART and chronic diseases follow-up clinics) and a supervisor (MPH student at Hawassa University) were recruited and training about the objectives and how to administer the questionnaire was given by the principal investigator. A pilot study on 5% of the sample was conducted prior to data collection. Data were collected through face-to-face interview and records review. During the data collection, safety measures were implemented to prevent the transmission of COVD-19 and other communicable diseases.

### Data analysis

Questionnaires filled were checked for completeness and then data were entered and analysed using SPSS-20 statistical software. Frequencies were calculated. Both bivariate and multivariable logistic regression models were used to identify associated factors. Variables significantly associated at less than or equal to 0.2 p-value in the bivariate logistic regression model were entered into a multivariable logistic regression model. The variables income and haemoglobin level high proportion of missing values and thus were excluded from the adjusted analysis. Odds ratios and their 95% confidence intervals (CIs) were computed and variables with p-value less than 0.05 and 95% CIs not crossing the null value were considered for reporting statistically significant associations.

### Ethics

A written ethical clearance was obtained from the institutional review board of Hawassa University, College of Medicine and Health Sciences on 25/06/2021. Reference number: IRB/215/13. A letter of support was obtained from CMHS, HU. Participants were informed about the purpose, benefit, risk and confidentiality of the information they provide. Patients were involved on a voluntary basis. Before starting the interview, informed verbal consent was obtained from respondents. Participants were informed that they have the right to withdraw from the study at any time in the course of data collection. Data were analysed anonymously.

## Results

A total of 187 PLHIV and 187 HIV negative people were enrolled. The median (IQR) age of PLHIV 53 (50–58) years and it was 59 (55–66) years for HIV negative controls. The median (IQR) CD4 count of PLHIV was 570 (393,724) cells/ml, the median (IQR) years since HIV diagnosis was 13 (11,15) and the median (IQR) years on ART was 12 (9,14). PLHIV were more likely to live in an urban centre than HIV negatives and also be poorer in terms of income. All demographic and clinical characteristics for study participants are shown in Table 1.

**Table 1:** Socio-demographic and clinical characteristics of the study participants.

Characteristics	PLHIV, n (%)	HIV-uninfected, n (%)	Total	P-value
Age, median (range)	53 (50, 80)	59 (50–88)	56 (50,88)	
CD4 count, median (IQR)	570 (58,3211)			
Years with HIV, median (IQR)	13 (11,15)			
Years on ART, median (IQR)	12 (9,14)			
Age				
50–58 years	142 (75.9)	87 (46.5)	229 (61.2)	<0.001
> 58 years	45 (24.1)	100 (53.5)	145 (38.8)	
Gender				
Men	91 (48.7)	113 (60.4)	204 (54.5)	0.02
Women	96 (51.3)	74 (39.6)	170, 45.5)	
Address				
Urban	180 (96.3)	115 (61.5)	295 (78.9)	<0.001
Rural	7 (3.7)	72 (38.5)	79 (21.1)	
Education				
No education	43 (23.0)	78(41.7)	121 (32.4)	<0.001
Has education	144(77.0)	109(58.3)	253 (67.6)	
Marital status				
Single life**	95 (50.8)	34(18.2)	129 (34.5)	<0.001
Married	92(49.2)	153(81.8)	245(65.5)	
Occupation				
No job or house wife	50 (26.7)	33 (17.6)	83(22.2)	<0.001
Farmer or other	13 (7.0)	48 (25.7)	61(16.3)	
Has job/pensioned	124 (66.3)	106 (56.7)	230 (61.5)	
Income*				
< 3000 ETB	76 (55.9)	71(40.6)	147(47.3)	0.007
> 3000 ETB	60 (44.1)	104 (59.4)	164(52.7)	
Years with comorbidities*				
< 8	19 (10.2)	128 (68.4)	147(39.3)	< 0.001
> 8 or more	168 (89.8)	59 (31.6)	227(60.7)	
Haemoglobin level				
< 12 gm/dl	15 (8.2)	10 (6.4)	25(7.3)	0.53
> 12 gm/dl	169 (91.8)	147 (93.6)	316(92.7)	
Admission history				
Yes	30 (16.0)	127 (67.9)	157(42.0)	< 0.001
No	157 (84.0)	60 (32.1)	217(58.0)	

ETB = Ethiopian Birr (* = 1 USD = 46.475 ETB); IQR = inter quartile range; PLHIV = people living with HIV; NCD = non communicable disease;

* = Years with HIV for PLHIV or years with the NCDs for controls, Single life

** = includes single, divorced and widowed

Table 2 shows the difference in the B-FIT components among PLHIV and controls. Ninety two (49.2%) PLHIV and 51 (27.3%) of controls had either low or high BMI. A majority of PLHIV, 120 (64.2%) in comparison to controls 58 (31.0%) had social problems. A higher proportion of PLHIV, 25 (13.4%) than controls, 10 (5.3%) had depression. Also, a high proportion of PLHIV 15 (8.0%) than controls 4 (2.1) reported a history of falling in the past 2 months. About three-fourth of cases (71.1%) and 111 (59.4%) of controls had right hand grip strength (RHGS) of below 30 kg. Nearly equal proportion of the cases 158 (84.5%) and controls 157 (84.0%) had problems in physical function. Majority of the cases, 120 (64.2%) and 58 (31.0%) of controls faced social function problems. The level of frailty and pre-frailty were higher among PLHIV than among controls. Except sensory and physical functions all other variables showed statistically significant differences among PLHIV and HIV-negative controls.

**Table 2:** Difference in frailty and its components measures among PLHIV and HIV negative controls.

Characteristics	PLHIV, n (%)	HIV-uninfected, n (%)	Total	P-value
BMI				
Low or high	92 (49.2)	51 (27.3)	143 (38.2)	<0.001
Normal	95 (50.8)	136 (72.7)	231 (61.8)	
Social function problem				
Yes	120 (64.2)	58 (31.0)	178 (47.6)	<0.001
No	67 (35.8)	129 (69.0)	196 (52.4)	
AD8 score				
Cognition problem	73 (39.0)	81 (43.3)	154 (41.2)	<0.001
No cognition problem	114 (61.0)	106 (56.7)	220 (58.8)	
Low BARTHEL score				
Yes	0 (0.0)	16 (8.6)	16 (4.3)	<0.001
No	187 (100.0)	171 (91.4)	358 (95.7)	
BDI score				
Yes (> 15)	25 (13.4)	10 (5.3)	35 (9.4)	0.008
No (0–14)	162 (86.6)	177 (94.7)	339 (90.6)	
Sensory function impairment				
No	155 (82.9)	147 (78.6)	302 (80.7)	0.29
Yes	32 (17.1)	40 (21.4)	72 (19.3)	
Falling in the past 2 months				
Yes	15 (8.0)	4 (2.1)	19 (5.1)	0.01
No	172 (92.0)	183 (97.9)	355 (94.9)	
Low TUG test				
< 13.5 seconds	6 (3.2)	0 (0.0)	6 (1.6)	0.01
> 13.5 seconds	181 (96.8)	187 (100.0)	368 (98.4)	
Slow gait speed				
< 0.8 m/s	1 (0.5)	13 (7.0)	14 (3.7)	0.001
> 0.8 m/s	186 (99.5)	174 (93.0)	360 (96.3)	
Low RHGS				
< 30 kg	133 (71.1)	111 (59.4)	244 (65.2)	0.01
> 30 kg	54 (28.9)	76 (40.6)	130 (34.8)	
Physical function problem				
No	29 (15.5)	30 (16.0)	59 (15.8)	0.9
Yes	158 (84.5)	157 (84.0)	315 (84.2)	
Social function				
No problem	67 (35.8)	129 (69.0)	196 (52.4)	< 0.001
Has problem	120 (64.2)	58 (31.0)	178 (47.6)	
Frailty				
Non frail	29 (15.5)	66 (35.3)	95 (25.4)	< 0.001
Pre-frail	141 (75.4)	110 (58.8)	251 (67.1)	
Frail	17 (9.1)	11 (5.9)	28 (7.5)	

BMI = body mass index; PLHIV = people living with HIV; NCD = non communicable disease; m/s = meter per second; TUG test = time up go test; BDI = becks depression inventory; RHGS = right hand grip strength

The median (IQR) frailty score was higher for PLHIV; 3 (2–3) compared to controls; 2 (1–3). Seventeen (9.1%) of PLHIV were frail compared to 11 (5.9%) controls. Among PLHIV, the proportion of frail was high for both genders, and urban dwellers compared to controls. For educated people the proportion of frail was also high compared to their counterpart. Among participants with low or high BMI, nearly similar proportion of cases and controls were frail. Among PLHIV with haemoglobin level of below 12 gm/dl, there was high prevalence of frailty; 3 (20.0%) (Table 3).

**Table 3:** Proportion of frail, pre-frail and non-frail among PLHIV and HIV-negative patients by various characteristics.

Characteristics	PLHIV, n (%)			HIV-uninfected, n (%)		
	Non-frail, n (%)	Pre-frail, n (%)	Frail, n (%)	Non-frail, n (%)	Pre-frail, n (%)	Frail, n (%)
Median frailty score	3.0 (0, 6)			2.0 (0, 6)		
All participants	29 (15.5)	141 (75.4)	17 (9.1)	66 (35.3)	110 (58.8)	11 (5.9)
Age						
50–58 years	26 (18.3)	106 (74.6)	10 (7.3)	47 (54.0)	36 (41.4)	4 (4.6)
> 58 years	3 (6.7)	35 (77.8)	7 (15.6)	19 (19.0)	74 (74.0)	7 (7.0)
Gender						
Men	19 (20.9)	66 (72.5)	6 (6.6)	38 (33.6)	65 (57.5)	10 (8.8)
Women	10 (10.4)	75 (78.1)	11 (11.5)	28 (37.8)	45 (60.8)	1 (1.4)
Address						
Urban	28 (15.6)	135 (75.0)	17 (9.4)	57 (49.6)	56 (48.7)	2 (1.7)
Rural	1 (14.3)	6 (85.7)	0 (0.0)	9 (12.5)	54 (75.0)	9 (12.5)
Education						
No education	5 (11.6)	33 (76.7)	5 (11.6)	10 (12.8)	58 (74.4)	10 (12.8)
Educated	24 (16.7)	108 (75.0)	12 (8.3)	56 (51.4)	52 (47.7)	1 (0.9)
Marital status						
Single	14 (14.7)	68 (71.6)	13 (13.7)	7 (20.6)	22 (64.7)	5 (14.7)
Married	15 (16.3)	73 (79.3)	4 (4.3)	59 (38.6)	88 (57.5)	6 (3.9)
Occupation						
House wife/No job	2 (4.0)	41 (29.1)	7 (14.0)	6 (18.2)	26 (78.8)	1 (3.0)
Farmer or other	2 (15.4)	9 (6.4)	2 (15.4)	4 (8.3)	35 (72.9)	9 (18.8)
Has job/Pensioned	25 (20.2)	91 (64.5)	8 (6.5)	56 (52.8)	49 (46.2)	1 (0.9)
Income						
< 3000 ETB	12 (15.8)	57 (75.0)	7 (9.2)	10 (14.1)	55 (77.5)	6 (8.5)
> 3000 ETB	12 (20.0)	47 (78.3)	1 (1.7)	52 (50.0)	47 (45.2)	5 (4.8)
BMI						
Low or high	6 (6.5)	74 (80.4)	12 (13.0)	3 (5.9)	40 (78.4)	8 (15.7)
Normal	23 (24.2)	67 (70.5)	5 (5.3)	63 (46.3)	70 (51.5)	3 (2.2)
Years with comorbidity*						
< 8	6 (31.6)	12 (63.2)	1 (5.3)	49 (38.3)	72 (56.2)	7 (5.5)
> 8 or more	23 (13.7)	129 (76.8)	16 (9.5)	17 (28.8)	38 (64.4)	4 (6.8)
Haemoglobin level						
< 12 gm/dl	0 (0.0)	12 (80.0)	3 (20.0)	3 (30.0)	6 (60.0)	1 (10.0)
> 12 gm/dl	29 (17.2)	127 (75.1)	13 (7.7)	53 (36.1)	85 (57.8)	9 (6.1)
Admission history						
Yes	3 (10.0)	24 (80.0)	3 (10.0)	39 (30.7)	78 (61.4)	10 (7.9)
No	26 (16.6)	117 (74.5)	14 (8.9)	27 (45.0)	32 (53.3)	1 (1.7)

BMI = body mass index; ETB = Ethiopian Birr (* = 1 USD = 46.475 ETB); PLHIV = people living with HIV; NCD = non communicable disease; gm/dl = gram per decilitre;

* = Years with HIV for PLHIV or years with the NCDs for controls

For PLHIV, the proportion of pre-frail was high among both genders, and urban dwellers. Among educated people the proportion of pre-frail was also high. A significant proportion of PLWH were found to be pre-frail compared to the controls; 141 (75.4%) vs 110 (58.8%). The proportions of pre-frail were higher for married and divorced PLHIV than controls. The number of PLHIV who were pre-frail with an income status of > 64.55 USD was very high compared to the controls. Nearly similar proportions of cases and controls were pre-frail among participants with low or high BMI. Among PLHIV with a haemoglobin level of below 12 gm/dl, there was high proportion of pre-frailty; 12 (80.0%). For patients with a history of admission, the proportion of frailty status was higher among PLHIV (Table 3).

The proportion of pre-frailty was lower in men 131 (64.2%) than in women 120 (70.6%). Prevalence of pre-frailty was higher for women participants in the age group of 50–58 than for men. Women PLHIV had a higher proportion of frailty than men (11 (11.5%) Vs 6 (6.6%)). For HIV uninfected controls the proportion of frailty was higher for men than women 10 (8.8%) Vs 1 (1.4%). For rural dwellers the proportion of pre-frailty was higher for women than men (19 (90.5%) Vs 41 (70.7%). The proportion of frailty among uneducated people was higher for men 10 (16.1%) than for women 5 (8.5%). The proportion of frail was higher for men farmers 11 (20.8%) than women 0 (0.0%). For participants earning at least 3,000 ETB per month, the proportion of frail participants among men was higher than among women 5 (4.9%) Vs 1 (1.6%). Of participants with a low or high BMI, nearly equal proportion of men and women were frail (11 (13.3%) Vs 9 (15.0%). Nearly equal proportions of men and women were frail when we consider both values of haemoglobin. Among participants with admission history, a higher proportion of men had frailty 10 (11.2%) Vs 3 (4.4%). (Table 4).

**Table 4:** Proportion of frail, pre-frail and non-frail by gender.

Characteristics	Men, n (%)			Women, n (%)		
	Non-frail, n (%)	Pre-frail, n (%)	Frail, n (%)	Non-frail, n (%)	Pre-frail, n (%)	Frail, n (%)
All participants	57 (27.9)	131 (64.2)	16 (7.8)	38 (22.4)	120 (70.6)	12 (7.1)
Age						
50–58 years	43 (36.1)	69 (58.0)	7 (5.9)	30 (27.3)	73 (66.4)	7 (6.4)
> 58 years	14 (16.5)	62 (72.9)	9 (10.6)	8 (13.3)	47 (78.3)	5 (8.3)
Address						
Urban	49 (33.6)	90 (61.6)	7 (4.8)	36 (24.2)	101 (67.8)	12 (8.1)
Rural	8 (13.8)	41 (70.7)	9 (15.5)	2 (9.5)	19 (90.5)	0 (0.0)
Education						
No education	7 (11.2)	45 (72.6)	10 (16.1)	8 (13.6)	46 (78.0)	5 (8.5)
Has education	50 (35.2)	86 (60.6)	6 (4.2)	30 (27.0)	74 (66.7)	7 (6.3)
Marital status						
Single life	8 (19.5)	26 (63.4)	7 (17.7)	13 (14.8)	64 (72.7)	11 (12.5)
Married	49 (30.1)	105 (64.4)	9 (5.5)	25 (30.5)	56 (68.3)	1 (1.2)
Occupation						
No job/House wife				8 (9.6)	67 (80.7)	8 (9.6)
Farmer	5 (9.4)	37 (69.8)	11 (20.8)	1 (12.5)	7 (87.5)	0 (0.0)
Has job/Pensioned	52 (34.4)	94 (62.3)	5 (3.3)	29 (36.7)	46 (58.2)	4 (5.1)
Income						
< 3000 ETB	14 (17.5)	58 (72.5)	8 (10.0)	8 (11.9)	54 (80.6)	5 (7.5)
> 3000 ETB	38 (37.3)	59 (57.8)	5 (4.9)	26 (41.9)	35 (56.5)	1 (1.6)
BMI						
Low or high	8 (9.6)	64 (77.1)	11 (13.3)	1 (1.7)	50 (83.3)	9 (15.0)
Normal	49 (40.5)	67 (55.4)	5 (4.1)	37 (33.6)	70 (63.6)	3 (2.7)
Years with comorbidities*						
< 8	31 (34.4)	51 (56.7)	8 (8.9)	24 (42.1)	33 (57.9)	0 (0.0)
> 8 or more	26 (22.8)	80 (70.2)	8 (7.0)	14 (12.4)	87 (77.0)	12 (10.6)
Haemoglobin level						
< 12 gm/dl	2 (15.4)	9 (69.2)	2 (15.4)	1 (8.3)	9 (75.0)	2 (16.7)
> 12 gm/dl	49 (29.3)	106 (63.5)	12 (7.2)	33 (22.1)	106 (71.1)	10 (6.7)
Admission history						
Yes	22 (24.7)	57 (64.0)	10 (11.2)	20 (29.4)	45 (66.2)	3 (4.4)
No	35 (30.4)	74 (64.3)	6 (5.2)	18 (17.6)	75 (73.5)	9 (8.8)

BMI = body mass index; ETB = Ethiopian Birr (* = 1 USD = 46.475 ETB); NCD = non communicable disease; gm/dl = gram per decilitre;

* = Years with HIV for PLHIV or years with the NCDs for controls; PLHIV = people living with HIV

HIV-status, age, place of residence, education, marital status, occupation, income, hemoglobin level and BMI showed an association with frailty in a bivariate analysis. However, in a multivariate analysis HIV-status, education, marital status, occupation, and BMI maintained significance in predicting frailty. The risk of frailty was higher among PLHIV compared to their counterparts (aOR 9.7; 95% CI 1.6–56.8). There was a lower risk of frailty among people aged 50–58 years (aOR 0.2; 95% CI 0.1–0.7) and an increased risk of frailty among uneducated people (aOR 5.2; 95% CI 1.5–18.2). Among singles, widowed or divorced there was a high risk of frailty (aOR 4.2; 95% CI 1.3–13.6). Farmers had a high risk of frailty (aOR 19.5; 95% CI 3.5–109.1). Participants with low or high BMI had a higher risk of frailty (aOR 47.3; 95% CI 13.8–161.9) (Table 5).

**Table 5:** Multinomial regression analysis for the risk factors of frailty status.

Characteristics	Pre-frail		Frail	
	cOR (95%CI)	aOR (95%CI)	cOR (95%CI)	aOR (95%CI)
Patient type, PLHIV	2.9 (1.8, 4.8)	4.2 (1.8, 9.9)	3.5 (1.5, 8.4)	9.7 (1.6, 56.8)
Age 50–58 years	0.4 (0.2, 0.7)	0.3 (0.1, 0.6)	0.3 (0.1, 0.7)	0.2 (0.1, 0.7)
Gender, Men	0.7 (0.5, 1.2)	0.6 (0.3, 1.3)	0.9 (0.4, 2.1)	1.3 (0.3, 5.9)
Residence, Urban	0.4 (0.2, 0.7)	0.4 (0.1, 1.2)	0.3 (0.1, 0.7)	0.6 (0.1, 4.2)
Education, no education	3.0 (1.7, 5.6)	2.2 (1.0, 4.9)	6.2 (2.4, 15.5)	5.2 (1.5, 18.2)
Marital status, single or others#	2.0 (1.1, 3.4)	1.1 (0.5, 2.3)	6.3 (2.5, 15.8)	4.2 (1.3, 13.6)
Occupation				
House wife	4.8 (2.2, 10.6)	2.6 (0.9, 7.2)	9.0 (2.7, 29.8)	5.0 (0.9, 27.8)
Farmers	4.2 (1.7, 10.4)	3.2 (1.0, 10,2)	16.5 (4.9, 55.3)	19.5 (3.5, 109.1)
Years with comorbidities* (> 8)	0.4 (0.2, 0.6)	0.7 (0.3, 1.4)	0.3 (0.1, 0.7)	0.8 (0.2, 3.1)
Low or high BMI	8.0 (3.8, 16.5)	11.3 (5.0, 25.8)	23.9 (8.2, 69.6)	47.3 (13.8, 161.9)

The reference is: Non frail; BMI = body mass index; PLHIV = people living with HIV; gm/dl = gram per decilitre; cOR = crude odds ratio; aOR = adjusted odds ratio;

* = Years with HIV for PLHIV or years with the NCDs for controls;

# single, widowed and divorced are together

Concerning pre-frailty variables like HIV-status, age, place of residence, education, marital status, occupation, income, years with the disease and BMI showed association with pre-frailty in unadjusted analysis. However, in the multivariate analysis only HIV-status, age, education, occupation and BMI showed an association with pre-frailty. The risk of pre-frailty was about four times (aOR 4.2; 95% CI 1.8–9.9) higher among PLHIV. In the age group 50–58 years the risk of pre-frailty was lower (aOR 0.3; 95% CI 0.1–0.6). Farmers (aOR 19.5; 95% CI 3.5–109.1) and people without formal education had an increased risk of pre-frailty (aOR 2.2; 95% CI 1.0–4.9). Participants with low or high BMI had a higher risk of pre-frailty (aOR 47.3; 95% CI 13.8–161.9) (Table 5).

There was an increased risk of low or high BMI among PLHIV. Compared to controls PLHIV had low measurement in the social dimension assessment (aOR 3.3; 95% CI: 1.8–5.9). Participants who lived for at least 8 years with their disease had increased risk poor record in the social dimension assessment, (aOR 2.2; 95% CI: 1.3–3.8). Patients with hemoglobin value of below 12 had a higher risk of depression (aOR 5.1; 95% CI: 2.0–13.1). Compared to HIV uninfected controls, PLHIV had a low risk of low hand grip strength, (aOR 0.5; 95% CI: 0.3–0.9) (Table 6).

**Table 6:** Associations between individual components of the B-FIT and clinical characteristics of the study participants.

Characteristics	BMI		Social dimension	
	cOR (95%CI)	aOR (95%CI)	cOR (95%CI)	aOR (95%CI)
Patient type, PLHIV	2.6 (1.7, 4.0)	2.4 (1.4, 4.2)	4.0 (2.6, 6.1)	3.3 (1.8, 5.9)
Years with comorbidities*, > 8	1.9 (1.3, 3.0)	1.1 (0.6, 1.9)	3.7 (2.3, 5.8)	2.2 (1.3, 3.8)
Admission history, Yes			1.6 (1.1, 2.4)	0.6 (0.4, 1.1)
	**Depression**		**Sensory function**	
Patient type, PLHIV	2.7 (1.3, 5.9)	1.6 (0.6, 4.1)		
Years with comorbidities > 8 years	2.8 (1.2, 6.6)	1.7 (0.6, 5.0)		
Haemoglobin, < 12 gm/dl	5.3 (2.1, 13.3)	5.1 (2.0, 13.1)	1.8 (0.7, 4.4)	1.7 (0.7, 4.4)
Admission, Yes			0.5 (0.3, 0.9)	0.6 (0.4, 1.0)
	**Low RHGS**			
Patient type, PLHIV	0.6 (0.4, 0.9)	0.5 (0.3, 0.9)		
Years with comorbidities*, > 8	0.8 (0.5, 1.2)	1.1 (0.6, 1.9)		
Haemoglobin, < 12 gm/dl	0.6 (0.2, 1.4)	0.6 (0.2, 1.5)		
Admission, Yes	0.8 (0.5, 1.2)	1.1 (0.7, 1.9)		

Note: The stated clinical characteristics did not show association with cognitive function and physical function, so it is omitted from this table

BMI = body mass index; PLHIV = people living with HIV; gm/dl = gram per decilitre; cOR = crude odds ratio; aOR = adjusted odds ratio;

* = Years with comorbidities = Years with HIV for PLHIV or years with the NCDs for controls

## Discussion

In this comparative cross-sectional study, high prevalence of frailty and pre-frailty were observed among PLHIV. The prevalence of pre-frailty among PLHIV was 38% in USA [9], 39.1% in Spain [4], 52.1% in another study in Spain [27] and 51.2% in Indonesia [13]. These estimates are lower than what we observed among our study participants. Frailty prevalence observed among PLWH in Hawassa was higher than the report from Spain, 4.4% [4], Indonesia 3.7% [13] and USA 6% [9]. Our finding is lower than the report from Spain, 15.4% [27] and South Africa, 19.4% [2]. However, it is within the range prevalence report among rural South African older population, 5.4% to 13.2% [28].

We have limited understanding of frailty in this newly emergent, aging population of PLWH receiving long-term ART in SSA. Two previous studies reported prevalence of 3.4%-19.4%, with differing frailty definitions and inclusion criteria [2, 11]. In the current analysis, using the B-FIT-2 metric, the prevalence of frailty and pre-frailty was 9% and 75% respectively, and these were higher in PLHIV compared to controls. However, a recent paper, also using the B-FIT-2 metric, showed a remarkably low prevalence of frailty of 0.68% among PWH over 50 years old in northern Tanzania [12]. The authors of the northern Tanzania study considered the high quality of care at the clinic as a possible contributor to the ultra-low prevalence of frailty. Population characteristics, treatment factors, socioeconomic variation and nutritional status differences might have also contributed to the observed differences in frailty prevalence. Majority, 76 (55.9%) of PLHIV in Hawassa were earning below 65 USD per month, 89.8% lived for over 8 years with comorbidities, 49.2% had low or high BMI scores, most of them had low HGS and physical/social function problems. Having different frailty assessment tools and a difference in approach to aging population could also have contributed to the observed difference between the reports. As people in SSA are not homogeneous, we suggest co-designing and validating the measurement in the context of SSA. Tools developed in high income countries may not work well for the population SSA which is why we used the BFIT-2, as it was developed to be used in the context of Africa. Probably we will need to do more studies across the region using this tool to define the real prevalence.

Concerning the components of frailty measures used in this study, having HIV infection was associated with lower HGS. This is consistent with previous reports [14, 29]. A study from Indonesia reported that the dominant frailty phenotype was weakness in HGS [13]. In South African PLHIV aged 50 years and over had an average HGS of 4.7 kg less than HIV-negative individuals [29]. In Jima Zone Ethiopia, adult PLHIV presented with lower HGS (−4.2 kg) at ART initiation than HIV negative patients [14]. Our finding is consistent with these reports.

Among our study population, PLHIV or controls with other chronic health conditions for at least 8 years had an increased risk of low value for engagement in social activities. Similarly, another study reported duration of disease as one of the factors causing social isolation [30]. Chronic pathology results in chronic symptoms which dramatically impair quality of life, physical and mental wellbeing and working capacity, also resulting in significant social care and economic burden [31]. This factor could contribute to the development of frailty.

One of the components of frailty measures in this study was depression and its prevalence was higher for patients with low hemoglobin levels. Similarly, other studies reported the association between low hemoglobin level and depression, particularly among the elderly [32, 33]. Despite it was not seen in the current study, other researchers reported the association between depression and frailty [34]. Controlling haemoglobin level may be important to minimize the risk of depression and then frailty among the elderly.

Our analysis showed that living with HIV increased the risk of both frailty and pre-frailty. Other reports from South Africa and China also showed similar results [1, 26]. HIV infection has also been associated with premature development of frailty, particularly in women [2]. In rural South African population, frailty rates were higher in women than in men [28]. Based on the study report by Blanco J-R and et al., gender was one of the independent predictors of frailty or pre-frailty [4]. Nearly equal proportions of men (7.8%) and women (7.1%) had frailty among our study participants. Though the proportion of pre-frailty was different in men (64.2%) and women (70.6%) among our study participants, there was no statistically significant difference by gender for both indicators. We suggest more attention be given to PLHIV for the timely identification and prevention of frailty and delivering early measures, which may reverse frailty or help in slowing its progress [35, 36].

Age is one of the main predictors of frailty and pre-frailty. The results of our study also showed the presence of association between pre-frailty/frailty and age. Many other reports confirmed the association between frailty and age [2, 28, 37]. As people get older, functions of the body diminish and thus they became frail. These findings suggest the importance of giving attention to older people to timely identify and prevent frailty.

Another factor that predicted both frailty and pre-frailty among our study participants was educational status. There is a report indicating that frailty was associated with lower educational levels [9], with a study by Sabine Rohrmann reporting that the actual prevalence rate of frailty or pre-frailty in a population could depend on socio-economic background and education [38]. This could be related to a lack of understanding amongst the less educated on how to minimize the risk of frailty; as a result, they acquire and live with the problem. Therefore, interventions targeted at socio-economic improvement may minimize the risk of frailty or pre-frailty among aging individuals regarding of HIV status.

Nutritional status is one of the key factors to prevent frailty and balanced nutritional status may reverse pre-frail status. HIV is a factor contributing to low nutritional status [39]. On the contrary, ART was associated with an increase in BMI [40] and PLHIV could have higher abnormal nutritional status than controls [41]. In the current study, people with low or high BMI had increased risk of both frailty and pre-frailty. A U-shaped association was observed between BMI and frailty confirming that not only underweight but also obesity is associated with frailty [42, 43]. Subjects who were underweight and those with high waist circumference and body fat mass presented a higher risk of frailty compared to normal subjects, while skeletal muscle mass was a protective factor for frailty in China [44]. To minimize the risk of frailty among the elderly, it is important having a normal body weight values.

A study from Australia reported that frailty was not associated with occupation among the elderly [45]. On the contrary an association between life-course occupational conditions and frailty was documented in a report by I. Iavicolia et al. [46]. In particular, intrinsically harder or manual occupations emerged as possible determinants of frailty manifestation and severity at older age [47]. Farmers across both groups had increased risk of frailty or pre-frailty among our study participants, which may be due to increased workload on them. Hard labour could be considered as a risk factor for frailty/pre-frailty regardless of HIV status. Thus, people with hard labour should be screened earlier so that we can identify when they are pre-frail, and timely intervention could be delivered to them to prevent frailty and provide rehabilitation.

Marital status was one of the factors which showed association with frailty among our study participants. Kojima G et al. in their study suggested the determinants of frailty were influenced by marital status [48]. A meta-analysis of cross-sectional data from 35 studies showed that unmarried individuals were almost twice more likely to be frail than married individuals [48]. Another study also reported that, unmarried men carried a higher risk of developing frailty than among married and widowhood and was also associated with the social frailty phenotype [49]. These findings suggest that among elderly, single life either being unmarried or being widowed can increase the risk of frailty. To minimize the risk of frailty among the elderly population provision of social support especially for those living alone may be helpful.

## Strengths and limitations

To the best of our knowledge this study is the first of its type in Southern Ethiopia that measured frailty among elderly PLHIV and HIV negative controls. The study has some limitations, as it is a cross-sectional study in which we collected data at a single point in time, reporting associations between variables may not be confirmatory. Though analysis of this study showed association between dependent and independent variables, for variables like occupation and BMI the interval estimates were not precise enough. Third limitation is missing of measuring some important variables such as the prevalence of those PLHIV who had a history of AIDS, and what the pre-ART CD4 count was. Both these parameters are known to be risk factors for frailty.

## Conclusion

This facility based cross-sectional study showed high median frailty score among PLHIV. The proportions of pre-frail and frail were higher among PLHIV. As studies are showing widely different prevalence of frailty in various settings, findings should be cautiously interpreted by health care providers when planning for care delivery. Various characteristics may contribute to the observed difference in prevalence. Despite the wide variation in prevalence, health care providers should be prepared to manage an increasing number of PLWH aging with frailty.

In this study, men had a lower level of pre-frailty than women, however this association was not statistically significant. Living with HIV, age, education, occupation and BMI predicted pre-frailty; while living with HIV, age, education, marital status, occupation, and BMI were factors that predicted frailty. There was an increased risk of low HGS, low or high BMI and low values in social dimension assessment among PLHIV. Living with comorbidity for a long period increased the risk of social isolation. We suggest the importance of early identification, prevention, and timely management of frailty and pre-frailty among PLHIV. Targeting those that are pre-frail and delivering interventions could be the best approach. Using screening tools, clinicians could identify them, provide comprehensive and timely care such as exercise, diet interventions, and social support for this aging people [50]. Understanding the biologic mechanisms and adapting proven geriatric principles of interdisciplinary care will improve the health and well-being of aging PLWH. We also suggest, conducting more studies across the SSA using the BFIT-2 tool could be helpful to define the real prevalence of frailty in the region.

## Supporting information

S1 File Questionnaire-typeset. (PDF)

S2 File The study tool. (PDF)

## References

[1] Yingying D, Haijiang L, Xing L, Frank YW, Yan VS, Vincent CM, et al. Higher Prevalence of Frailty Among a Sample of HIV-Infected Middle-aged and Older Chinese Adults Is Associated With Neurocognitive Impairment and Depressive Symptoms. The Journal of Infectious Diseases. 2017;215:687–92. doi: 10.1093/infdis/jix032 28329145

[2] Pathai S, Gilbert C, Weiss HA, Cook C, Wood R, Bekker LG, et al. Frailty in HIV-Infected Adults in South Africa. Journal of Acquired Immune Deficiency Syndromes. 2013;62(1):43–51. doi: 10.1097/QAI.0b013e318273b631 23018372 PMC3772340

[3] Thomas DB, Kenneth R. Frailty: a new vulnerability indicator in people aging with HIV. European Geriatric Medicine. 2019;10:219–26. doi: 10.1007/s41999-018-0143-2 34652747

[4] Blanco J-R, Barrio I, Ramalle-Go mara E, Beltran MI, Ibarra V, Metola L, et al. Gender differences for frailty in HIV-infected patients on stable antiretroviral therapy and with an undetectable viral load. PLoS ONE. 2019;14(5):e0215764. doi: 10.1371/journal.pone.0215764 31071105 PMC6508723

[5] Damani AP, Erlandson K, Kevin EY. Frailty in HIV: Epidemiology, Biology, Measurement, Interventions, and Research Needs. Curr HIV/AIDS Rep. 2016;13(6):340–8. doi: 10.1007/s11904-016-0334-8 27549318 PMC5131367

[6] Lewis EG, Coles S, Howorth K, Kissima J, Gray W, Urasa S, et al. The prevalence and characteristics of frailty by frailty phenotype in rural Tanzania. BMC Geriatr. 2018;18(1):283. doi: 10.1186/s12877-018-0967-0 30445919 PMC6240208

[7] Lewis E, Whitton L, Collins H, Urasa S, Howorth K, Walker R, et al. A brief frailty screening tool in Tanzania: External validation and refinement of the B-FIT screen. 2019. doi: 10.1007/s40520-019-01406-0 31811571

[8] Rockwood K, Song X, MacKnight C, Bergman H, Hogan DB, McDowell I, et al. A global clinical measure of fitness and frailty in elderly people. CMAJ. 2005;173(5):489–95. doi: 10.1503/cmaj.050051 16129869 PMC1188185

[9] Erlandson KM, Kunling W, Susan LK, Robert CK, Ronald JE, Babafemi T, et al. Association Between Frailty and Components of the Frailty Phenotype With Modifiable Risk Factors and Antiretroviral Therapy. The Journal of Infectious Diseases. 2017;215:933–7. doi: 10.1093/infdis/jix063 28453849 PMC5407051

[10] Marco R, Charles C. Characteristics of Frail Patients in a Geriatric-HIV Program: The Experience of an Urban Academic Center at One Year Follow-Up. Journal of the International Association of Physicians in AIDS Care. 2011;10(3):138–43. doi: 10.1177/1545109711399658 21502439

[11] Cournil A, Eymard-Duvernay S, Diouf A, groupe d’étude de la cohorte A. Vieillissement osseux et syndrome de fragilité à 10 ans de traitements ARV au Sénégal. Bulletin de la Société de pathologie exotique. 2014;107(4):238–40. doi: 10.1007/s13149-014-0350-4. 10.1007/s13149-014-0350-4 24615435

[12] Bristow C, George G, Hillsmith G, Rainey E, Urasa S, Koipapi S, et al. Low levels of frailty in HIV-positive older adults on antiretroviral therapy in northern Tanzania. Journal of NeuroVirology. 2021;27:58–69. doi: 10.1007/s13365-020-00915-3 33432552 PMC7921045

[13] Wulunggono W, Evy Y, Hamzah S, Edy RW, Youdiil O. Frailty among HIV-1 Infected Adults under Antiretroviral Therapy in Indonesia Current HIV Research. 2019;17(3). 10.2174/1570162X17666190828143947 PMC7061977 31456523

[14] Olsen MF, Kæstel P, Tesfaye M, Abdissa A, Yilma D, Girma T, et al. Physical activity and capacity at initiation of antiretroviral treatment in HIV patients in Ethiopia. Epidemiol Infect. 2015;143:1048–58. doi: 10.1017/S0950268814001502 25034136 PMC9507108

[15] Mark B. Frailty in people living with HIV. AIDS Res Ther. 2018;15 19. doi: 10.1186/s12981-018-0210-2 30445966 PMC6240180

[16] Dean AG, Sullivan KM, Soe MM. OpenEpi: Open Source Epidemiologic Statistics for Public Health, Version 2013 [2021/05/17].

[17] Gray WK, Paddick SM, Kisoli A, et a. Development and validation of the identification and intervention for Dementia in Elderly Africans (IDEA) study dementia screening instrument. J Geriatr Psychiatry Neurol. 2014;27:110–8. doi: 10.1177/0891988714522695 24578459

[18] Dewhurst F, Dewhurst MJ, Gray WK, et al. The prevalence of disability in older people in Hai, Tanzania. Age Ageing. 2012;41:517–23. doi: 10.1093/ageing/afs054 22516800

[19] Gray WK, Orega G, Kisoli A, et al. Identifying frailty and its outcomes in older people in rural Tanzania. Exp Aging Res. 2017;43:257–73. doi: 10.1080/0361073X.2017.1298957 28358296

[20] Emma GL, Louise AW, Harry C, Sarah U, Kate H, Richard WW, et al. A brief frailty screening tool in Tanzania: external validation and refinement of the B‑FIT screen. Aging Clinical and Experimental Research. 2020;32:1959–67. doi: 10.1007/s40520-019-01406-0 31811571

[21] Galvin JE, a.l.et. The AD8, a brief informant interview to detect dementia. Neurology. 2005;65:559–64. 16116116 10.1212/01.wnl.0000172958.95282.2a

[22] Heslin JM, Soveri PJ, Winoy JB, Lyons RA, Buttanshaw AC, Kovacic L, et al. Health status and service utilisation of older people in different European countries. Scand J Prim Health Care. 2001;19(4):218–22. doi: 10.1080/02813430152706710 11822643

[23] Podsiadlo D, Richardson S. The timed "Up & Go": a test of basic functional mobility for frail elderly persons. J Am Geriatr Soc. 1991;39(2):142–8. 1991946 10.1111/j.1532-5415.1991.tb01616.x

[24] Fried LP, Tangen CM, Walston J, Newman AB, Hirsch C, Gottdiener J, et al. Frailty in older adults: evidence for a phenotype. J Gerontol A Biol Sci Med Sci. 2001;56(3):M146–56. Epub 2001/03/17. PubMed doi: 10.1093/gerona/56.3.m146 . 11253156

[25] Jackson-Koku G. Beck Depression Inventory. QUESTIONNAIRE REVIEW. Occupational Medicine. 2016;66:174–5. 26892598 10.1093/occmed/kqv087

[26] Beck AT, Steer RA, Brown GK. BDI-II: Beck Depression Inventory Manual. San Antonio: TX: Psychological Corporation; 1996.

[27] Branas F, Jimenez Z, Sanchez-Conde M, Dronda F, De-Quiros JCLB, Perez-Elias MJ, et al. Frailty and physical function in older HIV-infected adults. Age and Ageing. 2017;46:522–6. doi: 10.1093/ageing/afx013 28203694

[28] Pathai S, Gilbert C, Weiss HA, al et. Frailty in HIV-infected adults in South Africa. J Acquir Immune Defic Syndr. 2013;62(1):43–51. 23018372 10.1097/QAI.0b013e318273b631 PMC3772340

[29] Negin J, Martiniuk A, Cumming RG, Naidoo N, Phaswana-Mafuya N, L LM, et al. Prevalence of HIV and chronic comorbidities among older adults. AIDS Lond Engl. 2015;26(Suppl 1):S55–S63. 10.1097/QAD.0b013e3283558459 PMC4404756 22781177

[30] Hwu YJ. The impact of chronic illness on patients. Rehabil Nurs. 1995;20(4):221–5. doi: 10.1002/j.2048-7940.1995.tb01632.x 7617969

[31] Roca M, Mitu O, Roca I-C, Mitu F. Chronic Diseases—Medical and Social Aspects. REVISTA DE CERCETARE SI INTERVENTIE SOCIALA. 2015;49:257–75.

[32] Trevisan C, Veronese N, Bolzetta F, Sergi G. Low Hemoglobin Levels and the Onset of Cognitive Impairment in Older People: The PRO.V.A. Study. January 2016. Rejuvenation Research. 2016;19(6). 10.1089/rej.2015.1768 26778182

[33] Umegaki H, Yanagawa M, Endo H. Association of lower hemoglobin level with depressive mood in elderly women at high risk of requiring care. Geriatrics and Gerontology International. 2011;11(3):262–6. doi: 10.1111/j.1447-0594.2010.00672.x 21199233

[34] Soysal P, Veronese N, Thompson T, Kahl KG, Fernandes BS, Prina AM, et al. Relationship between depression and frailty in older adults: A systematic review and meta-analysis. Ageing Res Rev. 2017;36(78–87). doi: 10.1016/j.arr.2017.03.005 28366616

[35] Abbasi M, Rolfson D, Khera AS, Dabravolskaj J, Dent E, L. Xia. Identification and management of frailty in the primary care setting. CMAJ. 2018;190(38):E1134–E40. 30249759 10.1503/cmaj.171509 PMC6157492

[36] Travers J, Romero-Ortuno R, Bailey J, Cooney MT. Delaying and reversing frailty: a systematic review of primary care interventions. Br J Gen Pract. 2019;69(678):e61–e9. doi: 10.3399/bjgp18X700241 30510094 PMC6301364

[37] Payne CF, Wade A, Kabudula CW, Davies JI, Chang AY, Gomez-Olive FX, et al. Prevalence and correlates of frailty in an older rural African population: findings from the HAALSI cohort study. BMC Geriatrics volume. 2017;17(1):293. 10.1186/s12877-017-0694-y PMC5745732 29281995

[38] Rohrmann S. Epidemiology of Frailty in Older People. Adv Exp Med Biol. 2020;216:21–7. doi: 10.1007/978-3-030-33330-0_3 31894543

[39] Kharti S, Amatya A, Shrestha B. Nutritional status and the associated factors among people living with HIV: an evidence from cross-sectional survey in hospital based antiretroviral therapy site in Kathmandu, Nepal. BMC Nutrition 2020, volume 6: 2. BMC Nutrition. 2020;6(22). 10.1186/s40795-020-00346-7 PMC7294605 32549993

[40] Olawepo JO, Pharr JR, Cross CL, Kachen A, Olakunde BO, Sy FS. Changes in body mass index among people living with HIV who are new on highly active antiretroviral therapy: a systematic review and meta-analysis. AIDS Care. 2021;33(3):326–36. doi: 10.1080/09540121.2020.1770181 32460518

[41] Apornpong T, Han WM, Chattranukulchai P, Siwamogsatham S, Wattanachanya L, Gatechompol S, et al. Higher Proportion of Abnormal Nutritional Status Among Well-Suppressed HIV-Infected Elderly Asians Compared to HIV-Negative Individuals. AIDS Res Hum Retroviruses. 2020;36(7):590–6. doi: 10.1089/AID.2019.0285 32093485

[42] RietmanML, van-der-ADL, van-OostromSH, PicavetHSJ, DolléMET, SteegHv, et al. The Association between BMI and Different Frailty Domains: A U-Shaped Curve?. J Nutr Health Aging 2018;22(1):8–15. doi: 10.1007/s12603-016-0854-3 29300416

[43] Watanabe D, Yoshida T, Watanabe Y, Yamada Y, Kimura M, Kyoto-KameokaStudyGroup. A U-Shaped Relationship Between the Prevalence of Frailty and Body Mass Index in Community-Dwelling Japanese Older Adults: The Kyoto-Kameoka Study. J Clin Med. 2020;9(5):1367. 32384756 10.3390/jcm9051367 PMC7290950

[44] Xu L, Zhang J, Shen S, Hong X, Zeng X, Yang Y, et al. Association Between Body Composition and Frailty in Elder Inpatients. Clin Interv Aging. 2020;15:313–20. Epub 2020 Mar 4. doi: 10.2147/CIA.S243211 32184580 PMC7061425

[45] Shi-Jynn Y, Gwini SM, Tembo MC, Ng BL, Low CH, Malon RG, et al. Frailty associations with socioeconomic status, healthcare utilisation and quality of life among older women residing in regional Australia. J Frailty Sarcopenia Falls. 2021;6(4):209–17. doi: 10.22540/JFSF-06-209 34950811 PMC8649863

[46] Iavicolia I, Lesoa V, Cesaribc M. The contribution of occupational factors on frailty. Geriatrics. 2018;75:51–8. 10.1016/j.archger.2017.11.010 29190544

[47] Trevisan C, Veronese N, Sergi G, al et. Marital Status and Frailty in Older People: Gender Differences in the Progetto Veneto Anziani Longitudinal Study. Journal of women’s health. 2016. 10.1089/jwh.2015.5592 26845424

[48] Kojima G, Walters K, Iliffe S, Taniguchi Y, Tamiya N. Marital Status and Risk of Physical Frailty: A Systematic Review and Meta-analysis. Journal of the American Medical Directors Association. 2020;21(3). Epub November 2019. doi: 10.1016/j.jamda.2019.09.017 31740150

[49] Olmo JG, Perxas LC, López-Pousa S, de-Gracia-Blanco M, Vilalta-Franch J. Prevalence of frailty phenotypes and risk of mortality in a community-dwelling elderly cohort. Age Ageing. 2013;42(1):46–51. doi: 10.1093/ageing/afs047 22454134

[50] Kwong J. Frailty in Adults With HIV: Identification, Assessment, and Management. The Journal for Nurse Practitioners. 2022;18:67e71.

