# Supplementary material for: Frailty status and associated factors among older PLHIV in Southern Ethiopia
Source: PLoS One. 2023 Apr 24;18(4):e0284376. doi: 10.1371/journal.pone.0284376 (PMC10124866; doi:10.1371/journal.pone.0284376)
Supplement: S2 File — (PDF) [file pone.0284376.s002.pdf]

Hawassa University, School of Public Health

Consent for participation.

Date ______________ Name of data collector ______________ Name of supervisor _______

This is a 30 – 40 minutes interview guide which is prepared for a study entitled "Frailty status and associated factors among PLHIV in Southern Ethiopia". The purpose of the study is to measure the prevalence of frailty and associated factors among elderly PLHIV and HIV non infected patients. You are selected to participate in the study as you are currently receiving ART or non-communicable disease care at HUCSH. It is your right not to participate in the study. However, your participation and genuine response is important to improve the quality of life of elderly people with frailty. Your name or any identifier will not be recorded and data will be kept confidential and used only for the study purpose. The findings of this research will be disseminated in aggregate. Do you agree to participate in the study? Yes/No. If responds yes the participant will be included in the study.

I. Socio-demographic and anthropometry related questions

1. Gender of the interviewee M _____ F _____,
2. When is your date of your birth in Eth. calendar (D/M/Y) ___________,
3. Where do you live? a. Rural ____ B. Urban ____
4. Educational status of the interviewee (the highest level achieved) ____________,
5. Marital status of the interviewee a. single b. married c. separated d. widowed e. other specify ______________________
6. Occupation of the interviewee a. house wife b. Daily laborer c. employed d. Merchant e. Other specify _____________,
7. How much is your household monthly income? _____________ Eth. Birr
8. Weight of the participant_________ kg , Height of the participant ________ meter,

II. Clinical and behavioral characteristics related questions

1. When were you diagnosed to have HIV? Write the date (D/M/Y) _________,
2. How long have you been on ART? Write the months/years ____________,
3. Patients' most recent CD4 count? ______date (D/M/Y) ___________, (record review)
4. Patients' most recent viral load level? __________date (D/M/Y) ___________,

5. Patients’ most recent HB level? ___________ date (D/M/Y) _____________

III. Cognitive function assessment (Coding used for IDEA cognitive screen questions)

1. Do you have problems with judgment (e.g. problems making decisions, bad financial decisions, and problems with thinking)? A. Yes ____ B. No ____

2. Are you less interested in hobbies/activities than before? A. Yes ____ B. No ____

3. Do you repeats the same things over and over (questions, stories, or statements)? A. Yes ____ B. No ____

4. Do you have trouble in learning how to use a tool, appliance, or gadget (e.g., computer, microwave, and remote control)? A. Yes ____ B. No ____

5. Do you forgets correct month or year? A. Yes ____ B. No ____

6. Do you have trouble in handling complicated financial affairs (e.g., balancing check book, income taxes, and paying bills)? A. Yes ____ B. No ____

7. Do you have troubles remembering appointments? A. Yes ____ B. No ____

8. Do you face daily problems with thinking and/or memory? A. Yes ____ B. No ____

9. Total AD8 SCORE _____________

IV. Physical function: using the Barthel Index

Table 1. The modified Barthel index: item definitions

Function/score	Description
Bowels	
0	Incontinent (or needs to be given enema)
1	Occasional accident (once a week)
2	Continent
Bladder	
0	Incontinent or catheterized and unable to manage
1	Occasional accident (maximum once per 24 h)
2	Continent (for more than 7 days)
Grooming	
0	Needs help with personal care: face, hair, teeth, shaving
1	Independent (implements provided)
Toilet use	
0	Dependent
1	Needs some help but can do something alone
2	Independent (on and off, wiping, dressing)
Feeding	
0	Unable
1	Needs help in cutting, spreading butter etc.
2	Independent (food provided within reach)
Transfers (bed/chair)	
0	Unable — no sitting balance
1	Major help (physical, one or two people), can sit
2	Minor help (verbal or physical)
3	Independent
Mobility	
0	Immobile
1	Wheelchair independent, including corners etc.
2	Walks with help of one person (verbal or physical)
3	Independent
Dressing	
0	Dependent
1	Needs help but can do about half unaided
2	Independent (including buttons, zips, laces etc.)
Stairs	
0	Unable
1	Needs help (verbal, physical, carrying aid)
2	Independent up and down
Bathing	
0	Dependent
1	Independent (bath, must get in and out unsupervised and wash self; shower, unsupervised/unaided)

Source: Royal College of Physicians/British Geriatrics Society (1992) [12].

V. **Depression assessment questions** (among the given options, which one best describes your situation)

1. 0 I do not feel sad. 1 I feel sad

2 I am sad all the time and I can't snap out of it. 3 I am so sad and unhappy that I can't stand it.

2. 0 I am not particularly discouraged about the future. 1 I feel discouraged about the future.

2 I feel I have nothing to look forward to.

3 I feel the future is hopeless and that things cannot improve.

3. 0 I do not feel like a failure. 1 I feel I have failed more than the average person.

2 As I look back on my life, all I can see is a lot of failures.3 I feel I am a complete failure as a person.

4. 0 I get as much satisfaction out of things as I used to. 1 I don't enjoy things the way I used to.

2 I don't get real satisfaction out of anything anymore. 3 I am dissatisfied or bored with everything.

5. 0 I don't feel particularly guilty 1 I feel guilty a good part of the time.

2 I feel quite guilty most of the time. 3 I feel guilty all of the time.

6. 0 I don't feel I am being punished. 1 I feel I may be punished.

2 I expect to be punished. 3 I feel I am being punished.

7. 0 I don't feel disappointed in myself. 1 I am disappointed in myself.

2 I am disgusted with myself. 3 I hate myself.

8. 0 I don't feel I am any worse than anybody else.

1 I am critical of myself for my weaknesses or mistakes.

2 I blame myself all the time for my faults.   3 I blame myself for everything bad that happens.

9. 0 I don't have any thoughts of killing myself.

1 I have thoughts of killing myself, but I would not carry them out.

2 I would like to kill myself.   3 I would kill myself if I had the chance.

10. 0 I don't cry any more than usual.   1 I cry more now than I used to.

2 I cry all the time now.   3 I used to be able to cry, but now I can't cry even though I want to.

11. 0 I am no more irritated by things than I ever was.

1 I am slightly more irritated now than usual.

2 I am quite annoyed or irritated a good deal of the time.   3 I feel irritated all the time.

12. 0 I have not lost interest in other people.

1 I am less interested in other people than I used to be.

2 I have lost most of my interest in other people.   3 I have lost all of my interest in other people.

13. 0 I make decisions about as well as I ever could.   1 I put off making decisions more than I used to.

2 I have greater difficulty in making decisions more than I used to.

3 I can't make decisions at all anymore.

14. 0 I don't feel that I look any worse than I used to.

1 I am worried that I am looking old or unattractive.

2 I feel there are permanent changes in my appearance that make me look unattractive

3 I believe that I look ugly.

15. 0 I can work about as well as before.

1 It takes an extra effort to get started at doing something.

2 I have to push myself very hard to do anything. 3 I can't do any work at all.

16. 0 I can sleep as well as usual.

1 I don't sleep as well as I used to.

2 I wake up 1-2 hours earlier than usual and find it hard to get back to sleep.

3 I wake up several hours earlier than I used to and cannot get back to sleep.

17. 0 I don't get more tired than usual. 1 I get tired more easily than I used to.

2 I get tired from doing almost anything. 3 I am too tired to do anything.

18. 0 My appetite is no worse than usual. 1 My appetite is not as good as it used to be.

2 My appetite is much worse now. 3 I have no appetite at all anymore.

19. 0 I haven't lost much weight, if any, lately. 1 I have lost more than five pounds.

2 I have lost more than ten pounds. 3 I have lost more than fifteen pounds.

20. 0 I am no more worried about my health than usual.

1 I am worried about physical problems like aches, pains, upset stomach, or constipation.

2 I am very worried about physical problems and it's hard to think of much else.

3 I am so worried about my physical problems that I cannot think of anything else.

21. 0 I have not noticed any recent change in my interest in sex.

1 I am less interested in sex than I used to be.

2 I have almost no interest in sex. 3 I have lost interest in sex completely.

22. Total BECHS depression SCORE ______________

VI. Morbidity assessment questions

1. What is the main reason of coming today to this hospital? ______________________

2. Do you have any of the following diseases diagnosed by a physician?

**Disease**	**Yes**	**No**	**Disease**	**Yes**	**No**
Arthritis			Stroke		
BPH			Epilepsy		
Cancer			Stroke		
Cardiac pathology			Dementia		
Cataract			Depression		
Chronic renal failure			Other mental diseases		
Respiratory pathology			HIV		
GI pathology			TB		
Diabetes mellitus			Anemia		
Hypertension			Other specify		

Sum of co-morbidities ____________

VII. Sensory functions, poly pharmacy, social support and self-reported health assessment questions

1. Do you have a hearing problem? A. Yes ____ B. No ____
2. In the last 30 days, how much difficulty did you have in seeing and recognizing an object at arm's length or in reading?

   a. no difficulty, b. mild difficulty, c. moderate difficulty, d. cannot do
3. In the last 30 days, did you face difficulty in seeing and recognizing a person you know across the road (i.e. from a distance of about 20 meters)?

   a. no difficulty, b. mild difficulty, c. moderate difficulty, d. cannot do
4. Do you use any regular medication, including traditional medicines and over the counter medications? A. Yes ____ B. No ____
5. What is it? ______________________________
6. How many types of medication do you use _______
7. How much problem do you have joining in community or other social activities? A. None, b. mild, c. moderate, d. severe, e. extreme/cannot do
8. How much problem do you have in maintaining a friendship?'

   A. None, b. mild, c. moderate, d. severe, e. extreme/cannot do
9. How is your health?' a. very poor, b. poor, c. neither good nor poor, d. good, e. very good
10. Do you consider yourself currently ill? A. Yes ____ B. No ____

VIII. Mobility related assessment questions

1. Did you have a falling incident the last 2 month 1. Yes 2. No
2. How many times did you fall in the last 2 months? _______________ times
3. Timed-up-and-go (TUG) test _______________ seconds
4. Gait speed over 4.5 meters _____________ m/s
5. Mean Hand grip strength (HGS)/3 measurements RT hand _______ LT hand ________

Thank you
